# PRO-FIT-CARE study: the feasibility assessment of a pilot online exercise intervention for persons living with obesity and female infertility

**DOI:** 10.3389/fspor.2024.1332376

**Published:** 2024-05-07

**Authors:** K. P. Wadden, N. Hollohan, T. Furneaux, R. Maher, C. M. Barrett, D. Fuller, F. Basset, D. Murphy, S. Murphy, S. Healey, E. McGowan, L. K. Twells

**Affiliations:** ^1^ School of Human Kinetics and Recreation, Memorial University of Newfoundland, St. John’s, NL, Canada; ^2^ Faculty of Medicine, Memorial University of Newfoundland, St. John’s, NL, Canada; ^3^ College of Medicine, University of Saskatchewan, Saskatoon, SK, Canada; ^4^ Discipline of Obstetrics and Gynecology, Memorial University of Newfoundland, St. John’s, NL, Canada

**Keywords:** infertility, obesity, exercise, feasibility, cardiorespiratory fitness

## Abstract

**Introduction:**

Moderate-to-high physical activity participation is associated with a reduced risk of infertility. Yet, exercise interventions that target cardiorespiratory fitness, independent of weight loss, are lacking in obesity and female fertility research.

**Purpose:**

The primary objective of the PRO-FIT-CARE (PROmoting FITness for CArdiometabolic & REproductive Health) study was to assess the feasibility of a moderate-to-high-intensity online exercise program for persons with obesity and female infertility.

**Methods:**

Feasibility, safety, acceptability, and efficacy were assessed by examining: (1) recruitment and consent rate, (2) study retention, (3) adverse events, (4) participant satisfaction, (5) adherence, and (6) cardiorespiratory fitness.

**Results:**

Eleven of thirty-two women contacted agreed to participate in the program (34.4% consent rate). Eight participants (72.7%) completed the study. One musculoskeletal injury was reported. There was a 30% adherence rate based on prescribed exercise intensity (60%–80% of heart rate maximum). One of eleven participants attended 80% of the exercise intervention. Based on a weekly satisfaction survey, the program had an overall high level of satisfaction. Compared to sex and age normative data, post-intervention, two of eight participants improved their cardiorespiratory fitness percentile rank.

**Conclusion:**

The study highlights challenges with adherence to an online exercise program. While the program was safe and participants reported high levels of program satisfaction, approaches to improve adherence must be incorporated.

## Introduction

Obesity, defined as a body mass index (BMI) greater than or equal to 30 kg/m^2^, is associated with a threefold increased risk of subfertility and infertility in females (1, 2). Menstrual irregularities, ovulatory disorders, and endometrial pathology are frequently observed in females of reproductive age who live with obesity (3). Further, persons with obesity are more likely to experience miscarriage, pregnancy complications, and poor outcomes with artificial reproductive technology (4). In Canada, between 2005 and 2018, obesity rates increased in both sexes (5), and parallel to this trend, infertility rates in Canada have more than doubled since the early 1980s (6). An increase in the number of patients diagnosed with obesity and infertility has led to a surge in demand for fertility treatments (7) and exposure to the limited health and social resources available for this often stigmatized patient population (8).

Weight loss is often the first therapeutic intervention for patients diagnosed with infertility and obesity (9). This recommendation is supported by evidence that a decrease of 5%–10% in one's body weight aids in the resumption of ovulation in persons affected by obesity (10–14). The prioritization of weight loss in the reproductive health field has inhibited the advancement of other non-weight-centric interventions (15). Further, weight stigma within the health system negatively impacts the quality of care for people living with obesity. Patients living with obesity frequently report adverse health outcomes, medication non-adherence, mistrust of healthcare providers, and avoidance of medical care (16). Navigating a diagnosis of obesity and infertility presents challenges, such as the potential for increased financial burden, for example, due to a lower chance of live birth per cycle. Additionally, failed fertility treatments are known to significantly impact a person's mental health and marital relationships (4).

Engagement in moderate-intensity physical activity has been shown to positively impact ovulation rate, menstrual cycles, and metabolic pathways associated with fertility, independent of weight loss or body weight (15, 17). In a recent meta-analysis of epidemiologic studies, evidence suggests that compared to low levels of physical activity (e.g., less than 30-min per week), moderate-to-high amounts of physical activity significantly reduced the overall risk of infertility (18). In this meta-analysis of ten studies, where two-thirds of the analyzed relative risk were adjusted for factors such as obesity, Xie et al. showed adherence to the recommended guidelines of at least 150-min per week of moderate-intensity aerobic physical activity further lowers the infertility risk (18). Significant variations in exercise protocols and insufficient reporting of methodologies make it challenging to draw conclusions on the effectiveness of exercise-based interventions on fertility-related outcomes for persons living with obesity (19). For example, Rothberg et al. implemented a weight-loss intervention that included a progressive exercise component where sedentary participants were encouraged to progress to 280-min of moderate-intensity physical activity per week over a 16-week period (20). However, it remains uncertain whether the participants in the intervention group reached this high level of targeted activity (21). The most recent Obstetrics and Gynecology national guidelines recommend pregnant or postpartum persons aim for 150- to 300-mins of physical activity over each week during and after pregnancy. Additionally, during preconception, persons planning to conceive should strive for a minimum of 150-min of moderate physical activity per week (22). Nevertheless, there remains a significant gap in our understanding of how physical activity influences reproductive outcomes for women diagnosed with obesity and infertility. Further, what we know of exercise prescription in this population is heavily influenced by observational data derived from patients' self-reported physical activity levels or accelerometry data (17, 23). Thus, due to the unique challenges of this population, there is a need for interventional studies to discern findings from association studies and identify the specific exercise protocols that enhance the efficacy of and adherence to physical activity.

In disease-specific populations such as diabetes, shifting the focus from weight loss to metabolism has resulted in the development of innovative and efficacious methodologies to advance our understanding of “metabolically healthy obesity” (24). In these studies, the methodological approach focuses on components of exercise protocols (e.g., low vs. moderate-to-vigorous intensity exercise) to target cardiometabolic health outcomes (e.g., glycaemic control, insulin sensitivity, and cardiorespiratory fitness) (25, 26). Based on observational studies, the duration of time spent engaging in moderate-to-vigorous intensity exercise has been shown to have no adverse effects on outcomes related to fertility in persons with obesity (BMI >25 kg/m^2^) (23). Similar to diabetes research, research is warranted to explore cardiometabolic health indicators that elucidate the association between moderate-to-vigorous physical activity and fertility outcomes that may occur independently of weight loss. However, while the shift in methodological focus has significantly contributed to advancing the field of exercise prescription, there has been substantial debate surrounding the feasibility of prescribing moderate-to-vigorous-intensity exercise in clinical populations (27, 28).

The primary objective of the PRO-FIT-CARE (PROmoting FITness for CArdiometabolic & REproductive Health) study was to determine the feasibility of a moderate-to-high-intensity online exercise program for persons with obesity and experiencing female infertility. To do this, we assessed feasibility, safety, acceptability and efficacy based on measurement of: (1) recruitment and consent rate, (2) study retention, (3) adverse events, (4) participant satisfaction, (5) adherence, and (6) cardiorespiratory fitness.

## Methods

### Study design and ethics

A pre-experimental feasibility pretest post-test study design with one group was conducted. The intervention took place in a virtual, online environment from June 2021 to September 2021 during COVID-19 restrictions. Ethical approval was obtained from the Provincial Health Research Ethics Authority (#20200467). Participants provided informed consent to take part in the study.

### Participants and recruitment process

Thirty-two participants were recruited for the study through two recruitment strategies: (1) targeted social media groups (e.g., Facebook fertility support groups) and (2) physician referrals from the local fertility clinic. Inclusion criteria for enrolment included women: (1) between the ages of 18 and 45 experiencing infertility (i.e., inability to conceive after twelve months of trying) through either self-report or physician referral, (2) with a BMI >30 kg/m^2^, (3) who were not meeting the Canadian Physical Activity Guidelines for physical activity, and (4) who are willing to commit to an online group exercise program three days a week for 12-weeks. Women were excluded from the study if they were <18 or older than 45 years, had physical impairments limiting their ability to participate, or were unwilling to delay fertility treatment for 16-weeks.

### Screening procedure

A Kinesiologist screened all participants using the “Get Active Questionnaire” for eligibility to participate in the exercise testing and intervention. If participants answered “Yes” to a question on the Get Active questionnaire, they were required to obtain written consent from their physician to participate.

### Patient and public involvement

The study design was reviewed by four members of the public with recent lived experiences of participating in an exercise program for persons with obesity and infertility. Refinements to the exercise protocol were made based on their feedback and suggestions. For example, patient partners discussed a preference for group-based exercise sessions and flexibility in scheduling the sessions.

### Exercise intervention

In accordance with ACSM guidelines, the online exercise intervention adhered to a gradually progressive program (29), which took place over 12 weeks. Exercise interventions of twelve-week durations have been shown to improve cardiorespiratory fitness in females of reproductive age with obesity (30). Participants were expected to attend three 45-min exercise sessions per week. Two sessions were conducted live and required mandatory attendance during the scheduled time (supervised training), while the third session was live-coached but offered optional attendance or the flexibility for it to be completed at a different time (unsupervised training). A private Facebook group was created to encourage participation, provide social support, remind participants of upcoming training sessions, and, most importantly, provide links to the virtual exercise intervention. The Facebook group was initiated to create a communal, supportive environment while being limited to virtual platforms due to COVID-19 restrictions.

The sessions were instructed by female Kinesiologists. Participants were encouraged to keep cameras on for safety during home exercise sessions (e.g., form correction) and ensure their space was clear for safe movement. Participants were instructed to wear heart rate (HR) devices, including a chest strap and watch. Attendance was recorded through multiple methods. First, participants were encouraged to self-report the completion of supervised and unsupervised training sessions in a Google Form posted weekly in the Facebook Group. Second, participants' HR data was collected using a Polar A370 HR monitor (HRM; Polar Electro OY, Kempele, Finland), and HR recordings were synched to a secured mobile device with the Polar Flow App and then downloaded using the Polar Flow Web service that was date and time stamped. HR data was cross-referenced with the self-reported completion data to confirm attendance.

**Figure 1:**
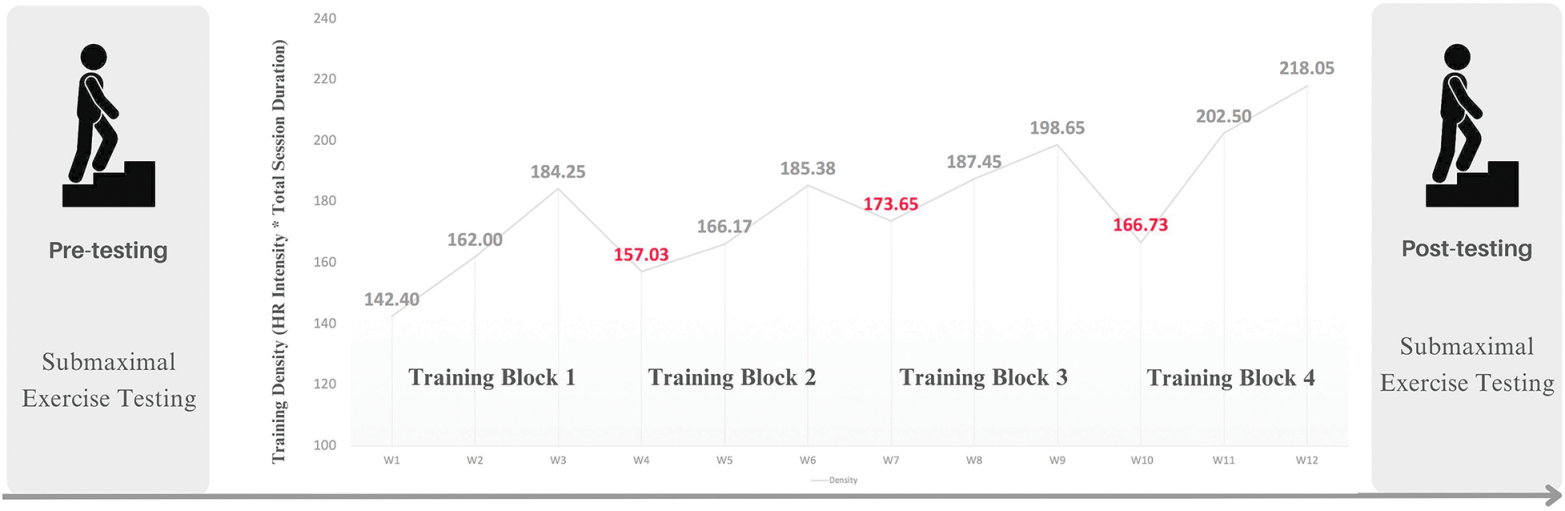
Research design and training blocks. Submaximal exercise testing, using the Modified Canadian Aerobic Fitness Test (mCAFT), was completed before and after the 12-week exercise intervention. The training density equalled the HR intensity multiplied by the total session duration (Interval duration + recovery time + warm up and cool down). The training density ranged from 117 to 275.4 s.

The exercise intervention was developed based on the general principles of training that include progressive overload, specificity, and recovery. Using a traditional periodization approach, the exercise invention was designed and delivered over four training blocks (i.e., mesocycles) consisting of three weeks of increasing load (31, 32). The training density (or workload) of each training block was determined by multiplying the total duration of the session (seconds) by the prescribed intensity (percentage of the HR maximum) (Figure 1) (see Supplementary Material 2 for a weekly description of the exercise intervention protocol). Further, the goal of each session varied, and the types of movements performed were to maximize chronic response and minimize overuse injuries (see Supplementary Material 3 for examples of prescribed movements). The first session of the week included light intervals focused on strength-based movements (e.g., body-weight squats). The second session targeted movements that yielded high-intensity exertion levels (e.g., fast marching) with minimal rest. The third session included movements that targeted moderate-intensity exertion levels with strength, stability, and cardiovascular-based movements (e.g., modified planks and jumping jacks). Each session included a ten-minute (600 s) warm-up and a ten-minute (600 s) cool-down period.

### Demographic and clinical data

Weight and height were self-reported by participants. Information from participants' medical charts at the fertility clinic and medical health records were extracted to characterize participants' physical and reproductive health status. The information included primary and secondary fertility diagnosis, and diagnosis of metabolic diseases, such as diabetes, cardiovascular disease, and infertility due to “male factor”.

The Godin-Shephard Leisure-Time Physical Activity Questionnaire was administered to determine eligibility and describe participants' physical activity levels before and after the exercise intervention (33). The Leisure-Time Physical Activity score was calculated to determine participants' physical activity level (34).

### Feasibility, safety and acceptability

According to the guidelines established by El-Kotob and Giangregorio for pilot and feasibility studies of physical activity interventions, the present study assessed the feasibility, safety, and acceptability of our exercise intervention with the goal of enhancing the rigour of future studies in the field (35). *Feasibility* was determined by measuring: (1) recruitment and consent rates, (2) outcome measure completion, and (3) adherence to protocols. Adherence to the protocol was based on achieving the prescribed HR intensity during the exercise session. HR data were collected with Polar wrist activity trackers and chest-strap HR monitors during exercise testing and sessions. Two HR measures were calculated to determine exercise adherence based on achieving the prescribed intensity: (1) average HR as a percentage of HR max per session and (2) maximum HR as a percentage of HR max per session. *Safety* was evaluated by monitoring and self-reporting adverse events, including falls or new health issues. *Acceptability* was assessed through two methods: (1) measuring the number of patients who completed ≥80% of the exercise intervention, and (2) participants' weekly satisfaction rating of various aspects of the program. The weekly satisfaction ratings were administered through Google Forms. Participants were asked to reflect on their week in the program and answer each question to the best of their ability. The majority of questions participants answered using a 5-point Likert scale, with “1” being “strongly agree” and “5” being “strongly disagree”. Participants were asked to rate their rating of perceived exertion (RPE) for each of the sessions they participated in that week.

### Intervention efficacy

To evaluate the efficacy of the exercise intervention, cardiorespiratory fitness was assessed using the Modified Canadian Aerobic Fitness Test (mCAFT) before and after the exercise intervention. Due to COVID-19 restrictions, participants performed the submaximal exercise testing virtually using the Zoom online platform. Participants were requested to have a support person present during the test. For the nine participants who lived within the metropolitan area, custom-made, two-step stairs that complied with the mCAFT testing criteria were delivered to participants' homes. The two participants who resided outside the metropolitan completed the test on the stairs of their homes. Participants were instructed through a five-minute warm-up period on how to use the steps. Following the warm-up and familiarization with the test protocol, participants began the test at stage one due to the sedentary behaviour of the participants. The HR at the end of each stepping stage was recorded. The participant continued to the next stage if the obtained HR was under 85% of the age-predicted HR maximum (220 - their age). Each stepping session lasts for three minutes. The support person measured systolic and diastolic blood pressure and recorded it using an automated sphygmomanometer before and after the test.

Participants predicted $\dot{V}O_{2\mathrm{Max}}$ was calculated from the following equation:

$$\dot{V}O_{2\mathrm{Max}}\;(\mathrm{ml}\cdot kg^{-1}\cdot \min^{-1})=\;[17.2+(1.29\;\times O_{2}\;\mathrm{cost}*)\;-(0.09\times \mathrm{wt})\;-(0.18\times \mathrm{age})]$$

where * represents the oxygen cost in ml · kg^–1^ · min^–1^ during the final stage of stepping. Wt = weight in kilograms. Age = years.

The calculated predicted $\dot{V}O_{2\mathrm{Max}}$ scores were compared to sex and age-matched normative-referenced percentile values (36).

### Statistical analysis

Categorical demographic and clinical variables were presented as frequencies and percentages. The %HR was calculated from the %HRmax [based on the age-predicted HRmax (220-age)] to determine the exercise intensity. The average %HR and peak %HR per session were calculated and reported as mean and standard deviation per training block for each session. Percentages were calculated for the weekly satisfaction survey based on a Likert scale.

For calculated $\dot{V}O_{2\mathrm{Max}}$, pretest and post-test values were calculated and reported as mean and standard deviation. To descriptively examine the impact of HR adherence and exercise session attendance on changes in cardiorespiratory fitness, a composite score was calculated by multiplying the average peak %HR by the number of sessions attended.

## Results

### Demographic and clinical data

Participants ranged in age from 28 to 42 years at the time of enrolment, with a mean age of 34 (SD = 3.74) years and resided in the Canadian province of Newfoundland and Labrador. At baseline, average anthropometric measures of height and weight were 1.63 m (SD = 0.074) and 108 kg (SD = 19.52, Range: 68–133.8 kg). The average BMI was 40.3 ± 4.54 kg/m^2^ and ranged from 32.5 to 46.2 kg/m^2^, which classified participants as a weight status of obese class III (37).

At the time of the study, data from medical charts of the participants (*n* = 10) showed that one participant was diagnosed with primary infertility and three with secondary infertility. Three participants had a polycystic ovarian syndrome (PCOS) diagnosis, three participants had a documented “male factor” diagnosis (in addition to female infertility), two participants were diagnosed with unexplained infertility, one participant was diagnosed with low ovarian reserve, one with oligomenorrhea, one with adenomyosis, and another with recurrent unexplained pregnancy loss (Table 1). Of the ten participants, some had multiple diagnoses.

**Table 1:** Demographic and clinical characteristics of participants.

Participants	*n* = 11/10^a^; *n* ± SD
Age (years)	34 ± 3.74
Pre-self-reported weight (kg)	108.0 ± 19.52
Pre-BMI	40.3 ± 4.54
Fertility diagnosis and gynecological conditions	*n*
Primary infertility	1
Secondary infertility	3
PCOS	3
Male factor	3
Unexplained infertility	2
Decreased ovarian reserve	1
Oligomenorrhea	1
Adenomyosis	1
Recurrent unexplained pregnancy loss	1
Past medical history of metabolic conditions	
Hypertension	2
Fatty infiltration of liver	1
Sleeve gastrectomy	1

^a^ One participant was not a fertility clinic patient, and their medical history was not included outside of age, weight, and BMI.

### Recruitment, consent rates and outcome measure completion

**Figure 2:**
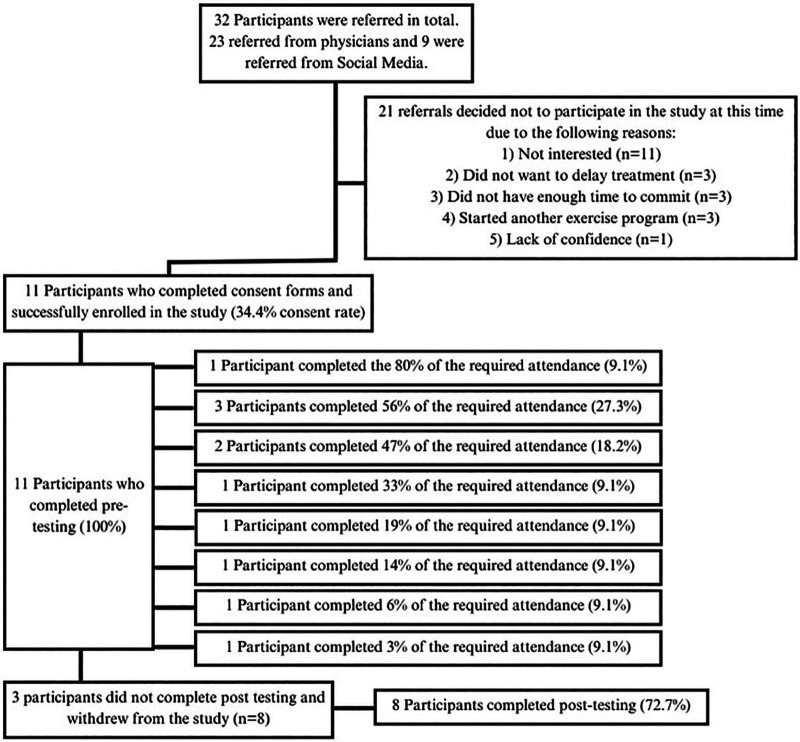
Recruitment flow diagram.

Thirty-two women were contacted to participate in the study. Eleven of 32 contacted women consented to participate in the study (34.4% consent rate). Eight participants (72.7%) completed the entire study. Participant recruitment is presented in Figure 2.

### Physical activity levels

According to the Leisure-Time Physical Activity score, eight out of eleven participants received scores below 14 before the intervention, indicating insufficient activity. Two participants scored between 14 and 23, indicating moderate activity, and one participant scored above 23, indicating sufficient activity. Following the exercise intervention, out of the seven participants who completed their questionnaires, five were classified as insufficiently active, one as moderately active, and one as sufficiently active.

### Heart rate adherence

For the total number of sessions attended by participants (*n *= 150), there was a 30% adherence rate for average %HR per session. However, when examining the peak HR achieved per session (averaged weekly), there was a 50% adherence rate for sessions attended by participants. Descriptively, the mean HR as a percentage of participants' age-predicted maximum increased over the first three training blocks [Training Block 1: 57.8% (SD = 5.0), Training Block 2: 62.1% (SD = 4.9), Training Block 3: 63.0% (SD = 4.7)], and decreased for the last training block [Training Block 4: 58.2% (SD = 5.5)] when the group attendance was low (mean # of sessions = 0.2, SD = 0.6). The peak HR% followed the same pattern (Table 2).

**Table 2:** Group data of the average HR as a percentage of HR Max across training blocks.

Training block	Week	Mean %HR	*SD*	Mean peak %HR	*SD*	Mean # of sessions attended	*SD*
1	TB 1, W1	55.2	*5.15*	70.3	*5.89*	1.8	*1.33*
	TB 1, W2	60.5	*4.85*	76.4	*6.59*	2.2	*1.66*
	TB 1, W3	57.7	*5.00*	75.8	*6.41*	1.4	*1.29*
	**TB 1 average**	**57**.**8**	**5**.**0**	**74**.**2**	**6**.**3**	**1**.**8**	**1**.**4**
2	TB 2, W4	59.0	*7.65*	76.7	*8.78*	1.4	*0.81*
	TB 2, W5	64.0	*3.63*	80.7	*5.61*	1.5	*1.04*
	TB 2, W6	63.4	*3.38*	82.2	*4.85*	1.0	*1.00*
	**TB 2 average**	**62**.**1**	**4**.**9**	**79**.**9**	**6**.**4**	**1**.**3**	**0**.**9**
3	TB 3, W7	63.2	*6.59*	82.2	*4.97*	1.4	*1.21*
	TB 3, W8	63.0	*4.83*	80.7	*4.57*	1.1	*1.51*
	TB 3, W9	62.7	*2.57*	83.2	*4.18*	0.8	*1.08*
	**TB 3 average**	**63**.**0**	**4**.**7**	**82**.**0**	**4**.**6**	**1**.**1**	**1**.**3**
4	TB 4, W10	64.4	*5.50*	83.9	*6.21*	0.6	*1.21*
	TB 4, W11	51.9	*N/A*	73.2	*N/A*	0.1	*0.30*
	TB 4, W12	N/A	*N/A*	N/A	*N/A*	0.0	*0.30*
	**TB 4 average**	**58**.**2**	**5**.**5**	**78**.**6**	**6**.**2**	**0**.**2**	**0**.**6**

Both the mean %HR and peak %HR per session per week were calculated. TB, training block; W, week; N/A, not applicable; %HR, percentage of heart rate maximum (i.e., 220 - Age); Peak %HR, highest heart rate recorded expressed as a percentage of heart rate maximum.

Italicized text represents the standard deviations for calculated means for %HR, peak %HR, and number of sessions attended.

Bolded text represents the TB Average Score for the calculated mean and standard deviation.

### Safety assessment, acceptability and satisfaction

**Figure 3:**
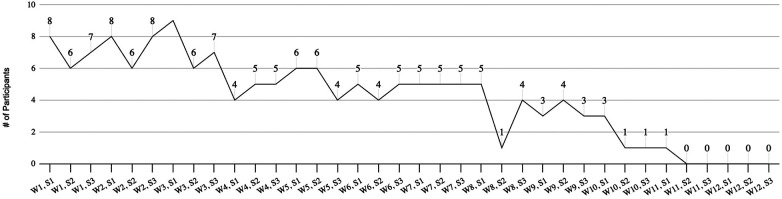
Group data of attendance of exercise sessions.

One adverse event was reported by a participant to the study's Kinesiologist and was recorded on the weekly survey. The adverse event was a musculoskeletal injury of the foot. The assessment of attendance reflected a low level of acceptance of the exercise intervention. One person (9.09%) achieved 80% of the required attendance deemed acceptable. Adherence to the program decreased as the 12-weeks progressed, with a significant decline in the fourth block of 4 weeks. Group attendance rates are summarized in Figure 3.

**Figure 4:**
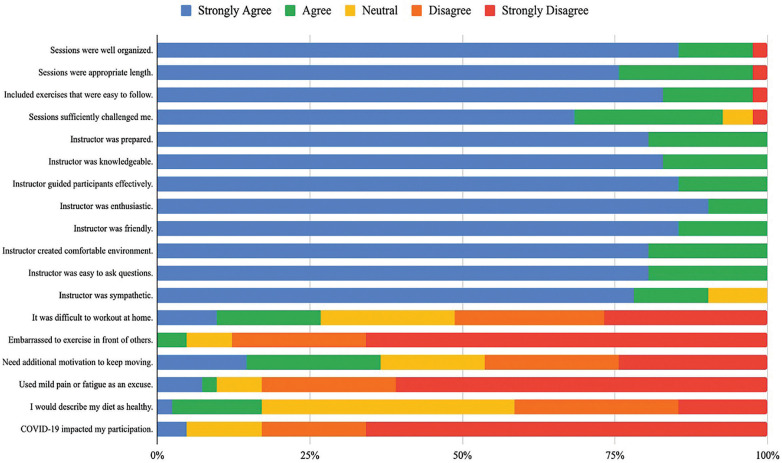
Group data of the weekly check-in survey: Likert scale results of participant satisfaction.

Based on results from the weekly satisfaction survey, participants reported overall high levels of satisfaction with the exercise intervention (Figure 4). The average response rate for the survey was 5.25, ranging from 2 to 8 responses per week. All positive statements regarding session structure and instructors had a majority of participants responding, “strongly agree”. Additionally, 51% of participants disagreed or strongly disagreed that completing the exercise sessions at home was difficult, and 87.9% disagreed or strongly disagreed that they were embarrassed to exercise in front of others. All participants (100%) responded “agreeing” or “strongly agreeing” that instructors created a comfortable environment during online classes. Participants did not report being impacted by COVID-19; however, the design and delivery of this intervention online were impacted by public health restrictions.

For the participants who responded to the weekly survey, the average RPE for all sessions was 14.68 (SD = 0.55), ranging from 11 to 19.

### Cardiorespiratory fitness

Before the exercise intervention (pre-test), the mean $\dot{V}O_{2}$ max was 26.0 ml · kg^−1^ · min^−1^ (SD = 4.54) with a range of 19.2–32.3 (Table 3). When comparing participants' cardiorespiratory fitness to normative-referenced percentile values for age and sex (31), all participants were below the 20th percentile. Following the exercise intervention (post-test), the mean $\dot{V}O_{2}$ max slightly improved, M = 27.1 ml · kg^−1^ · min^−1^, SD = 4.54, and the new range was 19.6–36.0. Of the eight participants who completed the post-exercise testing, two participants (ID 006 and 024) improved their percentile ranking and progressed from the 20th to the 50th and 10th to the 40th following the completion of the exercise intervention.

**Table 3:** Individual data for pre- and post-self-reported anthropometric data and $\dot{V}O_{2\mathrm{Max}}$ Max.

		Participant ID (*n* = 11)											Mean	*SD*
		001	005	006	008	009	013	017	019	024	027	031		
**Pre**	Weight (kg)	101.6	124.3	88.9	124.7	113.4	68.0	120.2	113.4	88.5	133.8	110.7	**108**.**0**	***19*** *.* ***52***
	Calculated BMI	41.0	44.2	32.6	43.1	41.6	32.5	42.8	42.9	36.8	46.2	39.4	**40**.**3**	***4*** *.* ***54***
	$\dot{V}O_{2}$ Max	25.5	23.5	32.3	22.7	25.4	27.8	32.0	23.5	25.1	19.2	29.3	**26**.**0**	***4*** *.* ***01***
	**VO_2_ Max percentile**	**<P5**	**<P5**	**<P20**	**<P5**	**<P5**	**<P5**	**<P20**	**<P5**	**<P10**	**<P5**	**<P20**	** **	*** ***
**Post**	Weight (kg)	102.3	124.1	82.7	*	*	68.0	120.2	*	88.5	129.1	*	**102**.**1**	***23*** *.* ***32***
	Calculated BMI	41.2	44.2	30.3	*	*	32.5	42.8	*	36.8	44.6	*	**38**.**9**	***5*** *.* ***78***
	$\dot{V}O_{2}$ Max	25.3	23.5	36.0^×^	*	25.4	27.8	28.8	*	30.2^×^	19.6	*	**27**.**1**	***4*** *.* ***89***
	**$\dot{V}O_{2\mathrm{Max}}$ Max percentile**	**<P5**	**<P5**	**<P50**	*	**<P5**	**<P5**	**<P5**	*	**<P40**	**<P5**	*		

Bolded text represents the VO_2_ Max percentile for each participant when compared to sex and age-matched normative-referenced percentile values pre- and post-intervention as well as the mean participant BMI and VO_2_ Max scores.

Bold-italics values represent the calculated standard deviation for the mean scores for participant BMI and VO_2_ Max.

* No data.

^×^ Improvement in $\dot{V}O_{2\mathrm{Max}}$ max.

The average adherence composite score (the average peak %HR * # of sessions attended) was 86.9 (SD = 60.29), ranging from 6.6 to 187.4. The two participants that improved their $\dot{V}O_{2}$ max percentiles values had the highest adherence composite score (187.5: post-test = <P50 of $\dot{V}O_{2}$ max; 147.81: post-test = <P40 $\dot{V}O_{2}$ max), demonstrating the highest adherence of exercise intensity and session attended.

## Discussion

Increased physical activity participation and adherence to average amounts of moderate-to-vigorous physical activity per week are associated with a reduced risk of infertility (18). However, due to current gaps in the delivery and assessment of non-weight-centric interventions for persons with an infertility diagnosis affected by obesity, there is a need to design, deliver, and evaluate the feasibility of an exercise intervention targeting cardiorespiratory fitness. The current pilot feasibility study showed low recruitment rates and adherence to a 12-week online group exercise intervention for women living with obesity and experiencing infertility. The low number of participants who completed pre-and post-testing and adhered to 80% of the exercise intervention limited our analysis. Nonetheless, due to the study's in-depth assessment of measures associated with feasibility, adherence, and efficacy, there are notable findings that can inform a more extensive study and future exercise interventions in this population.

In the present study, less than half of participants who contacted the research team consented to participate in the research study. While most participants completed the initial and follow-up assessment measures, of those who consented, adherence to the exercise intervention was low, with only one participant following the prescribed exercise sessions as recommended in the study protocol. Based on the consent rate and adherence to the exercise intervention, the present study design exhibited a lack of feasibility. This finding is reflected in other work investigating exercise interventions for persons living with obesity and experiencing infertility (38, 39). Nagelberg et al. studied the effects of a home-based exercise program that prescribed a progressive daily “step” count goal over four weeks on outcomes related to female infertility in an obese population with PCOS. While our exercise intervention design differed in exercise mode (e.g., group-based exercise, low-impact movements) and intensity (e.g., 60%–80% of HRmax) from the Nagelburg et al. study, similar to our study, only a third of the participants reached at least one weekly goal of 50% increase in their total “step” count.

In 2018, Kiel et al. published a pilot randomized controlled trial investigating the effects of a high-intensity 10-week exercise intervention performed on a treadmill three times weekly during supervised sessions. As only 18 participants consented to the study after four years, the study was concluded prematurely (21). The objective of the Kiel et al. study motivated the current study protocol, which was to prescribe moderate-to-high intensity rather than a general promotion of physical activity for weight loss. To expand the Kiel et al. protocol and improve the patient's desire to participate, we sought feedback from “patient partners” with experience participating in a physician-referred exercise program for persons with infertility diagnoses (40). The importance of group-based exercise with persons with similar lived experiences of living with obesity and experiencing infertility merged as a primary directive. Group-based exercise sessions have been shown to improve sources of motivation to participate in the exercise as participants feel a sense of relatedness and social connectedness to others in their group (41, 42). Due to COVID-19 restrictions, we delivered a virtual, group-based exercise intervention; however, the same degree of social connectedness may not have been attained in a virtual setting compared to in-person, impacting the level of adherence to the intervention. Further, while the home-based nature of the intervention may have removed barriers, such as travel time, it may have impacted accountability (43). Further, due to the extenuating circumstances surrounding the COVID-19 pandemic and its substantial impact on fertility services and patients' emotional and mental health (44), patients may have been less inclined to participate in research studies.

We explored participants' perceptions of the exercise intervention to understand factors that may impact feasibility (35). Based on results from the survey, there was overall high satisfaction, evidenced by the frequency of “strongly agree” ratings, which was the rating for approximately 75% of responses, for statements describing the quality and delivery of the exercise intervention. The ratings for statements regarding participation barriers were less conclusive; nonetheless, there was a tendency towards a greater number and frequency of participants rating “disagree” or “strongly disagree” for experiencing difficulties with completing the sessions from home, in front of others, needing additional motivators, and using mild pain or fatigue as a reason not to exercise. These findings suggested that the anticipated barriers to participation outlined in the survey were not necessarily those experienced by participants. For example, in a recent review by Hunter et al. (21) of randomized controlled trials that utilized physical activity in weight loss interventions, barriers to exercise were related to the safety of the neighbourhood and long working hours for persons living with obesity and infertility. Thus, barriers related to logistics, resources, and societal factors must be considered when developing and implementing an exercise intervention for persons with infertility.

Epidemiological studies indicate cardiorespiratory fitness is one critical indicator for reducing mortality risk (45–48). However, a limited understanding exists of how cardiorespiratory fitness, independent of BMI, contributes to reproductive health outcomes (49). Unfortunately, due to low numbers in the present study, it is difficult to comment on the effectiveness of the exercise intervention in improving cardiorespiratory fitness. However, descriptively, the two participants who improved their cardiorespiratory fitness percentile ranking most effectively completed the exercise intervention as measured by the intensity and frequency of exercise. Due to the many challenges with participation and adherence documented in the present study and in the research studies of others (38, 39), determining the effectiveness of cardiorespiratory fitness on fertility outcomes necessitates a focused effort on eliminating barriers to exercise participation and adherence.

### Limitations

There were several limitations to the present study. First, results were limited to descriptive statistics due to the small sample size. Thus, while our descriptive results are supported by other work in exercise and fertility research, there are significant constraints on the generalizability and utility of the present study's findings. Notably, the depth of conclusions regarding the effectiveness of the study's intervention is limited. Further, the study occurred during COVID-19 restrictions and the summer months (e.g., taking annual leave and hot temperatures and lack of air conditioning in the summer months), which may have impacted participants' motivation and priorities, and their ability to adhere to the exercise intervention.

Due to the COVID-19 restrictions, we experienced technical issues with menstrual cycle data collection through a mobile phone-based application. Specifically, the research team provided participants mobile phones to record menstrual cycle information. However, accessing the menstrual cycle application was challenging because of multiple security checks and trouble-shooting the device, which overburdened participants. This method may be feasible for future research if researchers provide in-person support or traditional methods, such as recording menstrual cycles via pen and paper, are used.

### Future directions

It is well-established that the assessment of cardiometabolic risk in the preconception phase is advantageous as individuals with poor cardiometabolic health are more susceptible to hypertensive disorders such as pre-existing hypertension, gestational hypertension, preeclampsia and gestational diabetes during pregnancy (50). Further, evidence from pregnancy literature provides a strong rationale for physical activity participation to improve biophysical markers (e.g., HDL-cholesterol, blood pressure, blood glucose and triglycerides), cardiorespiratory fitness and pregnancy outcomes (51). Future research must continue to explore the relationship between exercise-induced physiological changes and fertility outcomes, such as pregnancy viability and loss, that may occur independently of weight loss. Specifically, there is a crucial need to address the knowledge gap regarding how exercise regimens may improve live birth rates in individuals diagnosed with infertility and obesity (52).

Findings from epidemiological studies suggest associations between exercise (e.g., adherence to recommended guidelines of at least 150-min per week of moderate-intensity aerobic physical activity) and fertility outcomes, such as increased live birth or cumulative live birth [i.e., the preferred primary outcome for infertility research trials based on the “Improving the Reporting of Clinical Trials of Infertility Treatments” (IMPRINT) guidelines] (17, 23, 53). Yet, a recent study found that more than half of the women visiting a fertility clinic did not regularly participate in moderate or high-intensity exercise (50). There is an urgent need for the reproductive field to move beyond weight-centric approaches that recognize the limitations of a BMI measure and the potential benefits of exercise prescription for fertility care. More research is needed to characterize the cardiometabolic health of patients seeking fertility treatment and to determine the association between these measures and outcomes related to reproductive health in a broad anthropometric range of individuals. In conclusion, given the small sample size of the current study and issues related to recruitment and adherence, future research studies with larger sample sizes (e.g., through multisite recruitment strategies) and improved adherence methods (e.g., in-person sessions) will expand upon the findings of the present study.

### Conclusion

In conclusion, the present study design exhibited low *feasibility* based on a number of outcomes related to acceptability and adherence. The low level of acceptability and adherence was not unforeseen, given the online environment of the group-based sessions and the limited ability of the instructors to provide one-on-one feedback. Further, while the mode of exercise (e.g., low-impact body-weighted movements completed at home) was pragmatic, it was less controlled than, for example, cycling on a stationary bike or walking or running on a treadmill where the speed, resistance or incline can be manipulated in a laboratory setting. Future exercise and fertility research should investigate the feasibility of a hybrid approach of supervised one-on-one and group-based exercise to help improve participants' attendance and adherence to the intervention to an acceptable level, therefore allowing for an examination of the efficacy of exercise in improving outcomes related to fertility.

## Data Availability

The raw data supporting the conclusions of this article will be made available by the authors, without undue reservation.
