# Supplementary material for: PRO-FIT-CARE study: the feasibility assessment of a pilot online exercise intervention for persons living with obesity and female infertility
Source: Front Sports Act Living. 2024 May 7;6:1332376. doi: 10.3389/fspor.2024.1332376 (PMC11107087; doi:10.3389/fspor.2024.1332376)
Supplement: Supplementary file 1 [file Table1.docx]

Supplementary Material

**S1. Weekly Check-in Tool**

| **Questions on the program:** Answered using Likert Scale | This week, the program sessions were well organized. |
| --- | --- |
|  | This week, the program sessions were of appropriate length. |
|  | This week, the program sessions included exercises that were easy to follow. |
|  | This week, the program sessions sufficiently challenged me. |
| **Questions on the instructors:** Answered using Likert Scale | This week, the instructor was prepared. |
|  | This week, the instructor was knowledgeable. |
|  | This week, the instructor guided participants through exercises effectively. |
|  | This week, the instructor was enthusiastic. |
|  | This week, the instructor was friendly. |
|  | This week, the instructor created a comfortable workout environment. |
|  | This week, the instructor was easy to ask questions. |
|  | This week, the instructor was sympathetic. |
| **Questions regarding the participants’ experience:** Answered using the Likert Scale | This week, I felt it was difficult to exercise at home. |
|  | This week, I felt embarrassed to exercise in front of others. |
|  | This week, I need additional motivation to keep moving. |
|  | This week, I used mild pain or fatigue as an excuse. |
|  | This week, I would describe my diet as healthy. |
|  | COVID-19 has impacted my ability to participate in the program this week, ie. Lockdowns, motivation, vaccination. |
| **Addition Questions** | Additional comments on how you felt this week: |
| **Attendance and Intensity Questions** | How many sessions did you attend live? |
|  | What sessions did you attend live? |
|  | How many sessions did you do on your own time? |
|  | What sessions did you do on your own? |
|  | Using the below scale (Rating of Perceived Exertion from 6 to 20), please rate the intensity of (specific date) class. |
