# Supplementary material for: PRO-FIT-CARE study: the feasibility assessment of a pilot online exercise intervention for persons living with obesity and female infertility
Source: Front Sports Act Living. 2024 May 7;6:1332376. doi: 10.3389/fspor.2024.1332376 (PMC11107087; doi:10.3389/fspor.2024.1332376)
Supplement: Supplementary file 2 [file Table2.docx]

**Supplementary Material 2.** Exercise Intervention: Weekly Protocol

| **Week** | **Exercise Components** | **Day 1**  *Strength-Focus*  *Low-Intensity* | **Day 2**  *Aerobic-Focus*  *High-Intensity* | **Day 3**  *Strength/Aerobic*  *Moderate-Intensity* |
| --- | --- | --- | --- | --- |
| *Week 1* | **Intervals** | **2**  300s/150s  *60-65% HRR* | **2**  30s/30s **x6***  *80-85% HRR* | **1**  120s/60s **x5**  *70-75% HRR* |
|  | **Work-to-Ratio** |  |  |  |
|  | ***Heart Zone*** |  |  |  |
| *Week 2* | **Intervals** | **3**  300s/150s  *60-65% HRR* | **2**  30s/30s **x8***  *80-85% HRR* | **1**  120s/60s **x6**  *70-75% HRR* |
|  | **Work-to-Ratio** |  |  |  |
|  | ***Heart Zone*** |  |  |  |
| *Week 3* | **Intervals** | **3**  300s/135s  *60-65% HRR* | **2**  30s/30s **x10***  *80-85% HRR* | **1**  120s/60s **x 7**  *70-75% HRR* |
|  | **Work-to-Ratio** |  |  |  |
|  | ***Heart Zone*** |  |  |  |
| *Week 4* | **Intervals** | **3**  300s/150s  *60-65% HRR* | **2**  30s/20s **x6***  *80-85% HRR* | **1**  120s/45s **x5**  *70-75% HRR* |
|  | **Work-to-Ratio** |  |  |  |
|  | ***Heart Zone*** |  |  |  |
| *Week 5* | **Intervals** | **3**  300s/135s  *60-65% HRR* | **2**  30s/20s **x8***  *80-85% HRR* | **1**  120s/45s **x6**  *70-75% HRR* |
|  | **Work-to-Ratio** |  |  |  |
|  | ***Heart Zone*** |  |  |  |
| *Week 6* | **Intervals** | **4**  300s/120s  *60-65% HRR* | **2**  30s/20s **x10***  *80-85% HRR* | **1**  120s/45s **x7**  *70-75% HRR* |
|  | **Work-to-Ratio** |  |  |  |
|  | ***Heart Zone*** |  |  |  |
| *Week 7* | **Intervals** | **4**  300s/150s  *65-70% HRR* | **3**  30s/20s **x5***  *80-85% HRR* | **1**  120s/30s **x5**  *70-75% HRR* |
|  | **Work-to-Ratio** |  |  |  |
|  | ***Heart Zone*** |  |  |  |
| *Week 8* | **Intervals** | **4**  300s/120s  *65-70% HRR* | **3**  30s/20s **x7***  *80-85% HRR* | **1**  120s/30s **x6**  *70-75% HRR* |
|  | **Work-to-Ratio** |  |  |  |
|  | ***Heart Zone*** |  |  |  |
| *Week 9* | **Intervals** | **4**  300s/105s  *65-70% HRR* | **3**  30s/20s **x9***  *80-85% HRR* | **1**  120s/30s **x7**  *70-75%HRR* |
|  | **Work-to-Ratio** |  |  |  |
|  | ***Heart Zone*** |  |  |  |
| *Week 10* | **Intervals** | **4**  300s/105s  *65-70% HRR* | **3**  40s/20s **x5***  *80-85% HRR* | **1**  120s/30s **x5**  *70-75% HRR* |
|  | **Work-to-Ratio** |  |  |  |
|  | ***Heart Zone*** |  |  |  |
| *Week 11* | **Intervals** | **5**  300s/90s  *65-70% HRR* | **3**  40s/20s **x7***  *80-85% HRR* | **1**  120s/30s **x6**  *70-75% HRR* |
|  | **Work-to-Ratio** |  |  |  |
|  | ***Heart Zone*** |  |  |  |
| *Week 12* | **Intervals** | **5**  300s/60s  *65-70% HRR* | **3**  40s/20s **x10***  *80-85% HRR* | **1**  120s/30s **x7**  *70-75% HRR* |
|  | **Work-to-Ratio** |  |  |  |
|  | ***Heart Zone*** |  |  |  |

*Indicates that there was a prescribed 120s of rest between intervals. HRR = Heartrate Reserve; s = seconds.
