# Supplementary material for: PRO-FIT-CARE study: the feasibility assessment of a pilot online exercise intervention for persons living with obesity and female infertility
Source: Front Sports Act Living. 2024 May 7;6:1332376. doi: 10.3389/fspor.2024.1332376 (PMC11107087; doi:10.3389/fspor.2024.1332376)
Supplement: Supplementary file 3 [file Table3.docx]

**Supplementary Material 3.** Example of Exercise Intervention Movements

| **Dynamic Warm-Up Movements** | **Cool Down and Static Stretches** | **Strength-Based Movements** | **Aerobic-Based Movements** |
| --- | --- | --- | --- |
| Shoulder Rolls | Child’s Pose | Knee Plank | Jumping Jacks |
| Cat-Cow | Knees to Chest | Bird Dog | Cracker Jacks |
| Reach Throughs | Standing Hamstring Stretch | Glute Bridge | Standing Mountain Climbers |
| Arm Circles Forward/Backward | Shoulder Circles and Stretch | Body-Weighted Squat | Air Squats |
| Side-to-Side Steps | Half-Kneeling Hip Flexor | Single-Leg Balance | Air Boxing |
| Backwards Leg Kicks | Thread the Needle Hip Stretch | 3-Point Toe Touch | Sumo Squat Walk |
| Reach to Toes and Sky | Slow Marching on the Spot | Wall Push-up | Squat with Air Punches |
